# Poetic speech melody: A crucial link between music and language

**DOI:** 10.1371/journal.pone.0205980

**Published:** 2018-11-07

**Authors:** Winfried Menninghaus, Valentin Wagner, Christine A. Knoop, Mathias Scharinger

**Affiliations:** 1 Department of Language and Literature, Max Planck Institute for Empirical Aesthetics, Frankfurt, Germany; 2 Phonetics Research Group, Department of German Linguistics & Marburg Center for Mind, Brain and Behavior, Philipps-University Marburg, Marburg, Germany; Pennsylvania College of Health Sciences, UNITED KINGDOM

## Abstract

Research on the music-language interface has extensively investigated similarities and differences of poetic and musical meter, but largely disregarded melody. Using a measure of melodic structure in music––autocorrelations of sound sequences consisting of discrete pitch and duration values––, we show that individual poems feature distinct and text-driven pitch and duration contours, just like songs and other pieces of music. We conceptualize these recurrent melodic contours as an additional, hitherto unnoticed dimension of parallelistic patterning. Poetic speech melodies are higher order units beyond the level of individual syntactic phrases, and also beyond the levels of individual sentences and verse lines. Importantly, auto-correlation scores for pitch and duration recurrences across stanzas are predictive of how melodious naive listeners perceive the respective poems to be, and how likely these poems were to be set to music by professional composers. Experimentally removing classical parallelistic features characteristic of prototypical poems (rhyme, meter, and others) led to decreased autocorrelation scores of pitches, independent of spoken renditions, along with reduced ratings for perceived melodiousness. This suggests that the higher order parallelistic feature of poetic melody strongly interacts with the other parallelistic patterns of poems. Our discovery of a genuine poetic speech melody has great potential for deepening the understanding of the music-language interface.

## Introduction

Throughout most of its long history, poetry was part of the oral tradition and sung to genuine musical melodies [1]. This implies that many poems were, in all likelihood, not invented independently of musical melodies. With (Western) high-art poetry, however, this direct link between poems and melodies has been increasingly blurred. Modern poetry primarily constitutes a tradition of written texts that were and are read (silently or aloud), but no longer sung. It is also not known that authors wrote these poems with specific melodies in mind. To be sure, many poems impose higher prosodic regularity on language by virtue of implementing special metrical patterns. However, metrical stress and/or duration patterns alone are not known to predict genuine melodic pitch contours. Still, numerous authors of poems of the modern written tradition keep using the word “song” (German *Lied* or *Gesang*, French *chant*, etc.) in the title both of individual poems and of entire volumes of poetry; this applies at least across all major Western literary traditions. Similarly, the modern discourse on poetry has time and again emphasized musical properties of poems and a close link between poetry and music. It thus seems that, even though these poems are typically not sung, they still somehow convey an impression of *song-likeness* and *melodiousness*. It is precisely this persistent phantom of lyrical melody in the written tradition of poetry, which our study investigated. Our ambition was to show that this wide-spread emphasis on the song-likeness of written poetry is not just based on a vague comparison, let alone a mere phantom, but is actually reflective of a special, fully language-dependent, and objectively measurable type of repeated pitch and duration contours.

Recent brain imaging research has provided substantial evidence for such a close link between language and music processing in general ([2,3]). It is, however, still far from clear which formal features are actually shared by songs and *spoken* poems. For instance, both classical poetics and modern accounts have discussed similarities and differences between poetic and musical meter ([4,5]). Melodic features, however, have not been an issue in this context. 18^th^-century author Joshua Steele ([6]) is an exception to this rule: he proposed improvised musical (i.e., pitch-based) notations for poems, but did not relate these notations to perception. The present study is the first to extract in a systematic fashion the structure of pitch and duration sequences characteristic of individual poems from the acoustic sound envelope of various spoken renditions of these poems, and to correlate these measures with subjectively perceived melodiousness. In this effort, we exclusively focused on poems featuring a sustained rhyme, meter and stanza structure that was particularly predominant in 19th century Europe. This clearly is a limitation; however, the statistical measure we propose for poetic speech melody allows to control the hypothetical melody-effect for a potential dependence specifically on the prosodic hyper-regularity driven by meter.

Ratings for the perceived “melodiousness” of an utterance have repeatedly been used to capture perceptual features of spoken language (e.g. [7,8]). A “melodious” sound is often contrasted with a “monotonous” sound ([7]); by implication, a melodious sound envelope should be rich in variation. Moreover, there is broad consensus that pitch is indeed essential for both musical and speech-related prosody ([9,10]).

However, linguistic research on pitch has hardly gone beyond the phrase or sentence level. Recurrent pitch and duration contours characteristic of entire poems can only be captured by extending the analysis of such contours far beyond these narrow limits. Specifically, the analogy with musical songs suggests that the level of stanzas––which typically encompasses multiple individual phrases––is first and foremost the higher-order textual level of poems that is most likely to support recurrent melodic patterns. Notably, compositional arrangement in recurrent stanzas is also exactly what sets poetry of the type analyzed here most conspicuously apart from other genres of (literary) language ([11]). At the same time, conformity to a specific stanza pattern is a precondition for larger textual units to be sung to a given musical melody.

Accordingly, the arrangement in stanzas and the recognition of an *inherent language-based recurrence of pitch contours*––which we henceforth call “poetic speech melody”––should be intrinsically linked. We therefore expected that the stanza level should be particularly important for the construct of a poetic speech melody characteristic of entire poems. In order to be able to test this assumption, we included two additional units of analysis that are likewise important compositional building blocks of poems: all individual as well as rhyming lines only.

Pitch contours repeated over larger units of linguistic utterances are *prosodic (phonological) parallelisms* that implement structures of recurrence across these units. In this sense, poetic speech melody adds a novel dimension to the *parallelism hypothesis* of poetic language ([8,12,13,14]). This hypothesis stipulates that poetic language in general––whether found in traditional poems, free verse, political campaign slogans, commercial ads, or elsewhere––differs from ordinary language primarily by implementing multiple patterns of linguistically optional, yet perceptually salient, recurrence. The pertinent features include a great variety of often co-occurring phonological, syntactic, and semantic parallelisms (such as alliteration, assonance, anaphora, etc.), which are also frequently used in contexts other than rhymed and metered verses.

Most parallelistic features are limited to individual words, phrases and sentences, and hence *not continued* throughout entire texts (such as alliteration, assonance, anaphora, etc.). In contrast, meter, strophic structure and stanza-based recurrent pitch contours are higher order parallelistic features that imply a *continuous* parallelistic patterning across larger textual units. Non-continuous parallelistic features are likely to interact far less strongly both among each other and with continuous parallelistic features than the latter among each other. For instance, an alliteration can be removed or replaced by another one without necessarily affecting the overall metrical and strophic patterning. However, removing the ongoing metrical structure of poems should significantly affect their melodic structure, too, as musical melody cannot stay the same if its meter is altered or even completely eliminated. We therefore expected that the repetition of pitch contours across stanzas should be more sensitive to the experimental removal of poetic meter than to changes that primarily affect only individual words and syllables. At the same time, pitch contours of individual musical melodies can by no means be derived from their underlying meter only. Accordingly, we expected that poetic speech melody, too, cannot be reduced to a mere effect of meter.

Multi-parallelistic sentences and texts are linguistic analogues to multi-layered structures of symmetry, repetition and variation which are well-established as core features of the aesthetic appeal of both music and visual objects ([15,16,17,18]). Therefore, the focus on parallelistic structures in sentences and texts has the potential to allow for comparisons across aesthetic domains. Moreover, sensitivity to and priming through repetition is a general and highly important mechanism of human language perception and learning ([19]). Thus, the construct we propose relies on basic mechanisms of both aesthetic perception in general and language perception in particular. Notably, these mechanisms determine language use in general, including ordinary speech. This includes the near-regular distribution of stressed and unstressed syllables e.g. [20,21]) and the alternation of characters with large vs. small vertical spatial extent as well as the alternation of spaces between characters (e.g. [22]).

Our study specifically tested three hypotheses. Hypothesis 1 predicted for the set of 40 original poems that quantitative melodiousness scores obtained by autocorrelations of syllable pitch and duration should correlate with subjectively perceived melodiousness and hence be predictive of aesthetic appreciation. We extracted the melodic structure of these poems by identifying pitch and duration values for each syllable. This effectively allowed us to treat syllables in speech just like notes in music (for a similar approach, see [23] and [24]). In order to analyze the recurrence structure of pitch and duration, we subjected pitch and duration values to autocorrelation analyses ([25]). Autocorrelation is the cross-correlation of a time-series signal with itself at different time points; it has previously been used for detecting meter and melodic properties in music ([26,27,28]).

A second hypothesis informed our recourse to experimental modifications of the original poems. If poetic speech melody is indeed a higher order parallelistic structure of pitch and duration contours repeated across stanzas, it should be closely associated with the specific selection and combination of phonetic and prosodic building blocks implemented by the poet in the original text. Consequently, HYPOTHESIS 2 stipulates that increasing alterations and rearrangements of the wording should interfere with the hypothetical overarching melodic structure and reduce both objectively measured and subjectively perceived melodiousness.

If this hypothesis regarding different versions of the same poems holds true, it adds to the evidence for the existence of poetic speech melody that can be obtained through comparing different poems. Moreover, if modification-driven differences in melodiousness as measured by pitch and duration autocorrelations are largely convergent for the same poems across a variety of speakers, this would strongly support the notion that the construct of poetic speech melody is essentially speaker-independent.

Finally, we tested whether composers’ choices to put specific poems to music or not correlate with our measure of poetic speech melody. The affirmative option was our more speculative Hypothesis 3.

## Materials and methods

### Ethics statement

For all reported experiments, written informed consent was obtained from all participants, in accordance with the declarations of Helsinki. All experiments were approved by the Ethics Council of the Max Planck Society.

### Stimulus material

#### Poem corpus

We used a selection of 40 relatively unknown poems from the later 18th to the mid-20th century (written on average 160 years ago). The poems were originally collected and all of the other versions created for a study on how poetic diction influences emotional responses, specifically, feelings of joy, sadness, and being moved, and how these emotional responses correlate with aesthetic virtue attributions (specifically, beauty and melodiousness) as well as overall liking ([8]). All of the poems feature regular meter and either an ABAB (cross rhyme), AABB (pair rhyme), or ABBA (enclosed rhyme) scheme. On average, the poems consist of 4 ± 1 stanzas with 16 ± 4 lines and a total of 136 ± 40 syllables. Half of the poems were subsequently set to music, most prominently by romantic composers (information retrieved from the LiederNetArchive, http://www.lieder.net/lieder/index.html). Poem modification was done in four steps of removing parallelistic features, thus yielding five versions of each poem (see Table 1).

**Table 1:** Illustration of the poem modification on the first stanza of William Blake “Ah Sun-flower!”. The glossary column provides more detailed explanations of the specific modifications. The last column gives average number of syllables of the original and modified versions of the selected 40 poems.

Version	Properties	Example	Gloss.	Number of syllables [± SD]
A	Original	Ah Sun-flower! weary of time, Who countest the steps of the Sun: Seeking after that sweet golden clime Where the travellers journey is done.	Four verse lines, anapaestic trimeter (with verse-initial variation), cross-rhymed (abab)	136 ± 40
B	Meter, no rhyme	Ah Sun-flower! weary of time, Who countest the steps of the Sun: Seeking after that sweet golden place Where the travellers journey is through.	Rhyme removed by replacing the verse-final words in line 3 and 4	136 ± 38
C	Rhyme, no meter	Ah Sun-flower! weary of the time, Who countest all the steps of the Sun: You are seeking after that sweet golden clime Where the journey of the traveller is done.	Meter removed by adding syllables to break up the pattern	145 ± 42
D	No rhyme, no meter	Ah Sun-flower! weary of the time, Who countest all the steps of the Sun: You are seeking after that sweet golden place Where the journey of the traveller is through.	Integration of the modifications B and C	147 ± 41
E	No rhyme, meter and further parallelistic properties	Ah Sun-flower! weary of the time, Who countest all the phases of the Sun: Pursuing that sweet golden place Where the journey of the traveller is through.	Removal of the alliteration in “steps”/”sun” and the alliteration and assonance in “seek”/”sweet”	142 ± 42

Importantly, the modifications of the various parallelistic features did not affect several other features that are likewise characteristic of the type of poetry used in our study. For instance, a low degree of narrative content, unmediated evocations of highly personal and highly emotional speech situations, and the frequent addressing of an absent person/agent who is or was highly significant for the lyrical speaker are found across all versions of the poems. Moreover, non-parallelistic features of poetic diction (such as metaphor, ellipsis, etc.) were also kept as constant as possible. Finally, non-metered and non-rhymed poems account for a substantial share of 20th century poetry. For all these reasons, the modified versions that were relatively low in parallelistic features were also readily accepted as poems. Table 1 illustrates the steps of modifications we employed on our set of 40 German poems. In order to make these steps intelligible to a broader readership, we illustrate them on an English analogue which is based on the first stanza of the poem “Ah Sun-flower!” by William Blake. (For a detailed German example of all differences between versions A and E, see Supplementary Materials in Menninghaus et al., 2017.)

### Spoken renditions of the poem corpus

#### Professional speaker

The 200 stimuli overall (40 poems in five versions each) were recited by a professional speaker who is a trained actor, certified voice actor and speech trainer. The digital recordings (sampling rate 44.1 kHz, amplitude resolution 16 bits) were made in a sound-attenuated studio. The speaker was instructed to recite all poem versions with a relatively neutral expression and without placing too much emphasis on a personal interpretation Errors during reading were corrected by repeating the respective parts of the poems.

#### Synthetic voices

In order to obtain speaker-independent evidence of poem-based pitch and duration recurrences, we opted for several control conditions. One control condition involved computer-generated voices in a text-to-speech application (natively implemented in MAC OSX 10.11). All 5 versions of the 40 poems were synthesized using a male and a female voice at a syllable rate of ~4 syllables/s. We used the voices called ANNA (female) and MARKUS (male) in their standard-settings. The algorithm first translates text input into a phonetic script and then synthesizes each word using pre-recorded speech templates. Global prosodic features are applied by default and triggered by punctuation (e.g. question intonation is triggered by question mark). We decided to use these voices on the basis of the overall acceptable voice quality which was judged to be superior to many other text-to-speech synthesis applications, including some that allow for a more detailed control over acoustic-phonetic properties.

#### Nonprofessional speakers

Another control involved 10 nonprofessional native German speakers (4 males, 6 females, mean age 29 ± 7 years) who read a feasible subset of 8 original poems (version A) and 8 modified versions (version E) of these poems. Participants in this production study were recruited from the participant pool of the Max Planck Institute for Empirical Aesthetics and received monetary compensation. They were asked to read the 16 poems in randomized order, naturally and in a standing position in a sound-attenuated recording studio (sampling rate 44.1 kHz, amplitude resolution 32 bits). They were also asked to avoid strong expressivity in their renditions. Errors during reading were corrected by repeating the respective parts of the poems. Prior to acoustic analyses, the recorded poems were modified in order to match the written versions (replacement of erroneous passages, removal of non-text-based additions). All experimental procedures were ethically approved by the Ethics Council of the Max Planck Society and were undertaken with the written informed consent of each participant. On average, the recording session took about one hour per participant.

### Acoustic analyses

Our acoustic analyses focused on the primary acoustic cue of linguistic pitch, i.e., the fundamental frequency of sonorous speech parts (F0, [29,30]), and on syllable duration.

#### Preprocessing

Digitized poem renditions were automatically annotated using the Munich Automatic Segmentation system (MAUS), and annotation grids were imported into the phonetic software application PRAAT ([31]). Annotation was based on syllabic units; this was motivated by the observation that the syllable is a core linguistic unit in poetry ([32]) and the minimal unit in the prosodic hierarchy ([33]). The annotation was manually inspected and corrected by a trained phonetician and native speaker of German. Corrections included marking silent periods larger than 200 ms as pauses and shifting syllabic boundaries to zero crossings in order to arrive at consistent cuttings, as is usual in phonetic annotation.

In principle, the measure of recurrence we used is entirely independent of manual chunking or manual pre-processing. However, we decided for an approach that includes a “hand-made” control and fine-tuning of the exact syllable boundaries; we expected that this additional effort could improve the correlations between our statistical textual measure and the data for subjective perception. (A quantification of the degree to which our manual intervention actually improved the correlations was, however, beyond the scope of our paper.)

**Fig 1:**
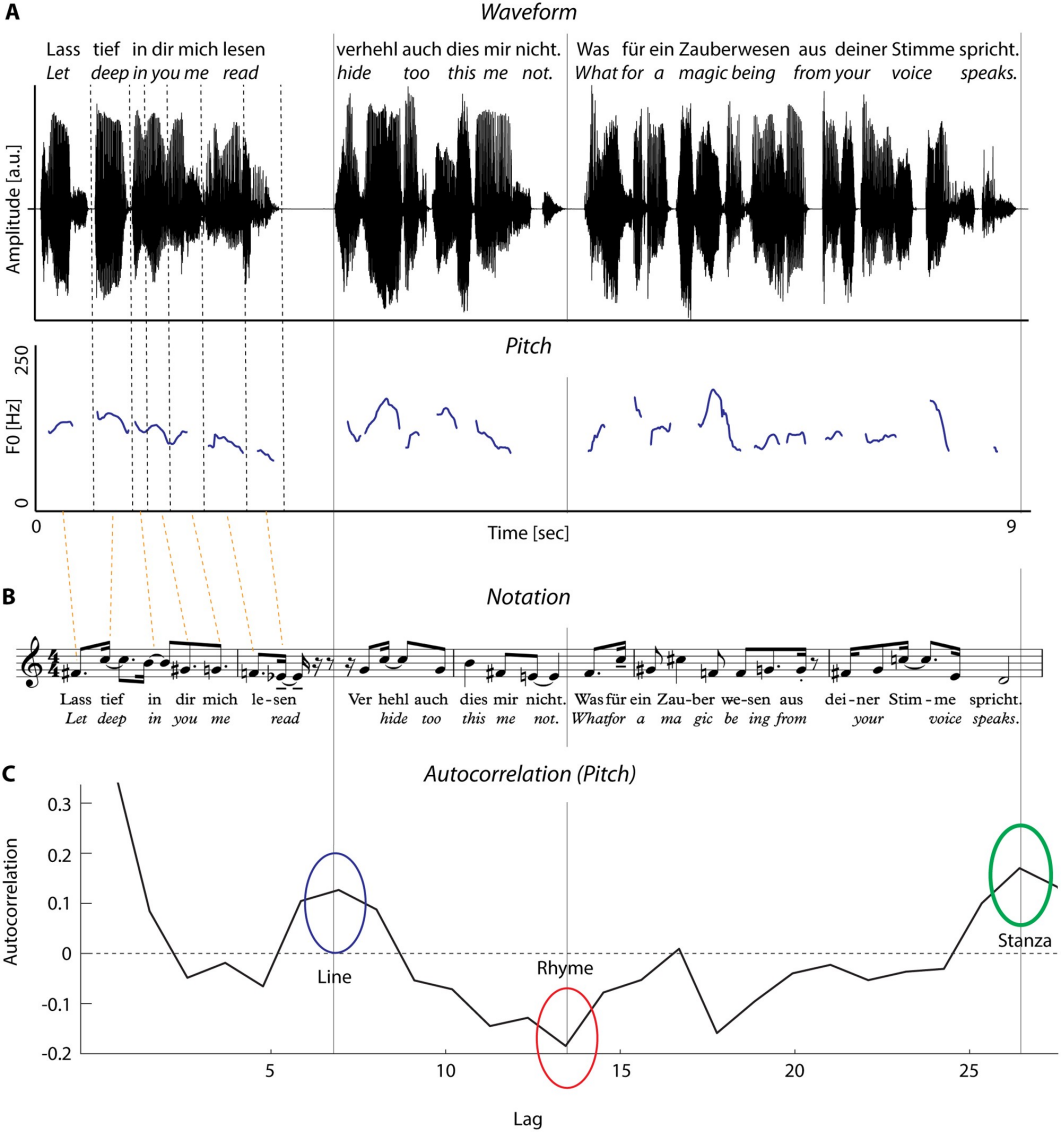
Illustration of the acoustic analysis workflow. A. Top: The digitized speech signal was annotated, using syllabic units (example poem: August von Platen [1814], Lass tief in dir mich lesen). Bottom: The pitch contour was obtained by a two-pass fundamental frequency (F0) estimation. From sonorous parts, the mean pitch at three measurement positions was calculated. B. Mean pitch values were mapped onto semitones, using the MIDI convention, and syllable duration was mapped onto musical length. For illustration purposes, the resulting notation was shifted two octaves up. C. Discrete pitch and duration values were subjected to autocorrelation analyses. Apart from an overall measure of autocorrelation strength, the study focused on autocorrelation values at lags that correspond to poetic structure, such as (all) individual lines, rhyming lines, and stanzas.

For all analyses, our syllable-based pitch estimation followed the approach in Hirst [34]. In a first pass, the fundamental frequency of the entire poem was calculated using an autocorrelation approach, with the pitch floor at 60 Hz and the pitch ceiling at 700 Hz. The 25% and 75% quartiles (Q25 and Q75) of pitches were identified and used for determining the pitch floor and ceiling in the second pass of fundamental frequency estimation. The second-pass pitch floor was 0.25 * Q25, while the pitch ceiling was 2.5 * Q75. Pitch extraction is illustrated in Fig 1. Averaged pitch and duration values for each speaker/voice are provided in Table 2.

**Table 2:** Basic acoustic properties of the recitations by the professional speaker, synthetic voices, and naïve (nonprofessional) speakers. Each speaker in the control group of 10 speakers produced a subset of 16 poems (8 original [A] versions, 8 modified [E] versions). The syllable rate is computed as the number of non-silent syllabic units per time unit.

Speaker	Mean syllable pitch [Hz ± SD]	Mean syllable rate [1/sec ± SD]	Number of poems [*n*]
**Professional (male)**	99 ± 6	3.0 ± 0.2	200 (40x A, B, C, D, & E, respectively)
**Synthetic (male)**	102 ± 2	4.1 ± 0.2	200
**Synthetic (female)**	173 ± 3	4.1 ± 0.2	200
**Naïve (female)**	196 ± 3	4.2 ± 0.3	16 (8x A, 8xE)
**Naïve (female)**	217 ± 5	2.9 ± 0.2	16
**Naïve (female)**	218 ± 6	3.3 ± 0.2	16
**Naïve (male)**	129 ± 3	3.1 ± 0.2	16
**Naïve (female)**	214 ± 7	3.4 ± 0.3	16
**Naïve (female)**	242 ± 3	2.9 ± 0.2	16
**Naïve (female)**	227 ± 5	3.6 ± 0.2	16
**Naïve (male)**	141 ± 2	3.6 ± 0.2	16
**Naïve (male)**	137 ± 3	4.0 ± 0.2	16
**Naïve (male)**	126 ± 2	3.2 ± 0.2	16

Following Patel et al. [23], we computed the mean pitch for each syllable across the three measurement positions beginning, middle, and end. In rare cases (<0.2% of all data points), pitch could not be determined and was interpolated based on the pitches of neighboring syllables. In addition to syllable-based pitch information, we also calculated the physical duration of each syllable. We excluded pauses from the pitch and duration analyses.

#### Pitch transformation

Pitch and duration values were discretized, using MIDI conventions, in order to arrive at a more music-analogous basis for subsequent pitch analyses. This implied that raw pitch values (in Hz) were transformed into semitones on the MIDI scales with numeric values ranging from 21 to 108, using the following formula:

$$d=69+12log_{2}\left(\frac{f}{440\;Hz}\right),$$ (1)

with *d* = MIDI pitch value and *F* = raw pitch (in Hz).

Syllable durations were mapped onto musical note durations, with the simplifying assumption that a whole note corresponds to 1 s. The smallest note duration values were mapped to a 16th note, which thus corresponded to a minimal syllable duration of 62.5 ms. For illustration purposes only, the MIDI pitch values were transposed two octaves up (see Fig 1B).

#### Autocorrelation analyses

We performed autocorrelation analyses on the time series of syllable/note pitches and syllable/note durations. The discrete autocorrelation *R* at lag *L* for the signal *y*(*n*) was calculated as

$$R\left(L\right)=\sum_{n\in Z}y\left(n\right)\bar{y}\left(n-L\right).$$ (2)

Autocorrelations were determined for lags *L* = 0 up to 90% of the length of the respective time series.

The significance of each autocorrelation value was estimated using a permutation analysis. For this purpose, autocorrelations were computed for 10,000 randomly shuffled syllable sequences. For each time lag, the absolute value of the autocorrelation was compared to the autocorrelation value for the original syllable sequence of each poem. If the autocorrelation value for the shuffled sequence was smaller than the autocorrelation value for the original sequence in more than 95% of all 10,000 permutations (α < 0.05), the hypothesis that the autocorrelation value of the original sequence equaled the autocorrelation value of the random sequence at the corresponding lag was rejected, and the autocorrelation value in question was then considered “significant”. Only significant autocorrelations were used for subsequent analyses.

For the main study, we determined the average distances (in number of syllables) between syllables of successive stanzas, rhyming lines only, and all verse lines, as we hypothesized that pitch and duration contours yield recurrent patterns across the main compositional building blocks of the poems (most prominently the stanza). For the modified poem versions from which rhymes were removed, distances (in number of syllables) between rhyming lines were determined based on where the rhyming word would have been found, had it not been replaced by a non-rhyming counterpart. Subsequently, we determined the mean autocorrelation value of each poem rendition for these three textual units (all individual lines, rhyming lines only, stanzas).

### Melodiousness rating data

Ratings of subjectively perceived melodiousness (given on a 7-point scale) were collected for all poem versions (A-E). Because our hypothesis considers the original version A as the most melodious one and we were interested in the hypothetical decrease of perceived melodiousness relative to this version, we consistently used version A as anchor version against which we separately compared all other versions.

#### Participants and procedure

Procedure and experimental setup was similar to the study reported by ([8]). Overall, 320 students (224 women and 96 men) participated (80 per the individual comparisons of version A with versions B, C, D, and E), with a mean age of 23.6 years (SD = 4.3, min = 18, max = 42). Each participant listened to two versions (original and one of the modified versions) of four poems recited by the professional speaker and was subsequently asked to rate these two versions on several 7 point-scales capturing emotional responses (positive and negative affect, sadness, joy and being-moved) and dimensions of aesthetic evaluations (melodiousness, beauty, and liking). For the present study, we exclusively focus on the melodiousness ratings.

#### Results

**Fig 2:**
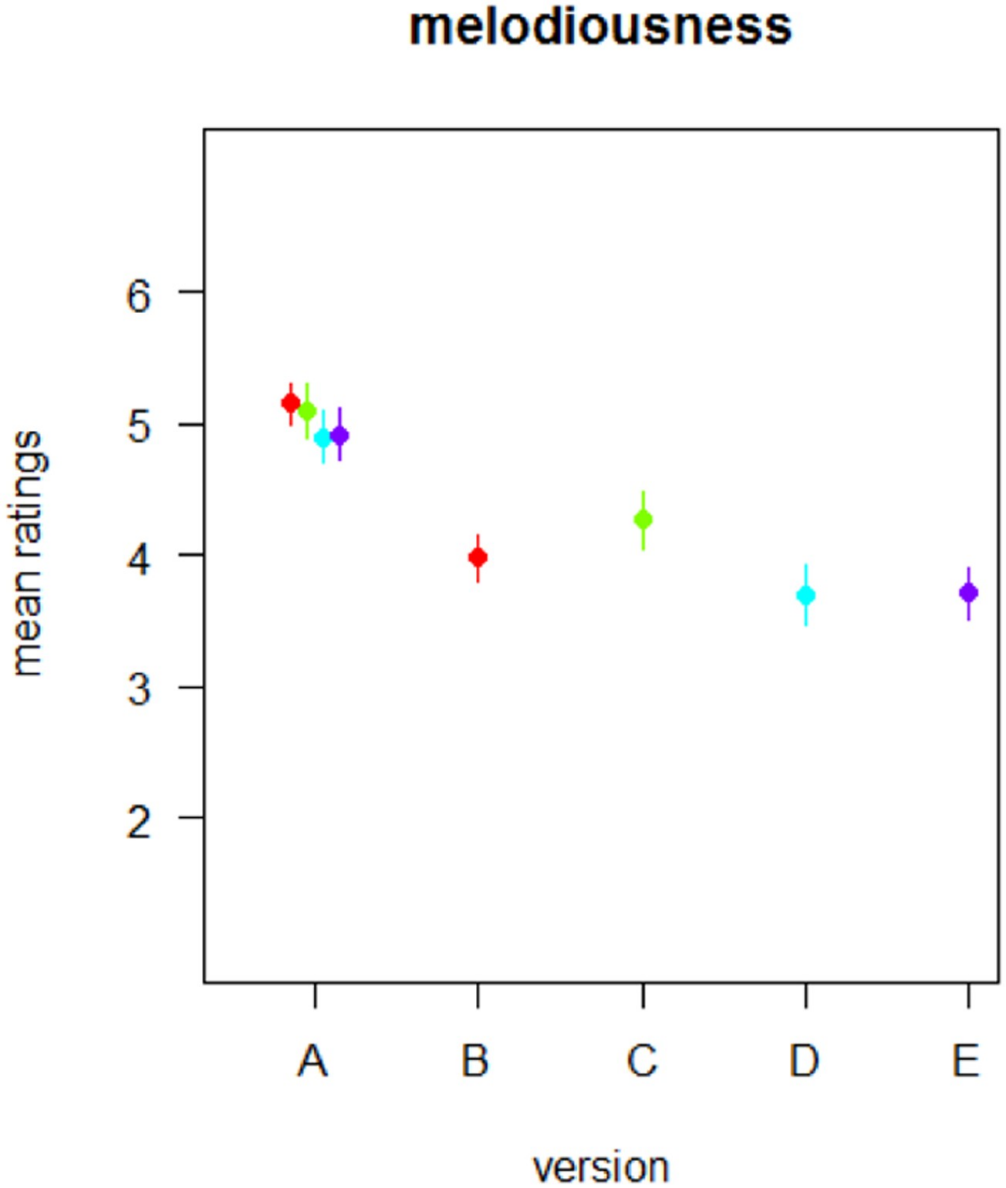
Mean melodiousness ratings for all poem versions. Notably, melodiousness ratings for the original poems (version A) did not differ between experiments.

The mean melodiousness ratings obtained for the original version (A) across the four different data sets did not significantly differ from one another (*F*(3,117) = 2.05, *p* = 0.110; see Fig 2). We therefore collapsed the ratings for version A across the four data sets.

### Statistical analyses

#### Correlation analyses

Correlation analyses were all based on Spearman’s rank correlation coefficients (carried out in the statistical software package R, Version 3.4, The R Foundation, Vienna, 2017).

#### Analyses of variance

We report all results as a mixed-effect ANOVA with *F*-values that were estimated by the lmerTest package ([35]). Post hoc analyses were calculated based on the multcomp package in R ([36]) and consisted of Bonferroni-adjusted *t*-tests with *z*-transformed *t*-values.

#### Correlations

For the dependent variable *mean autocorrelation* (for the three textual units of line, rhyme, and stanza) we calculated separate models for (a) the 200 renditions by the professional speaker and the two synthetic voices and for (b) the 16 renditions by the 10 nonprofessional speakers. All models included *poem* as a random variable and the fixed effects *poem version* (A to E for the professional speaker and computer voice models, and A vs. E for the nonprofessional speaker model), *acoustic measure* (pitch or duration), and *speaker* (professional speaker vs. the two synthetic voices for model (a) and 10 nonprofessional speakers for model (b), as well as all possible interactions). Finally, the models also included the fixed effect *textual unit* (all lines, rhyming lines, stanzas).

In order to further examine the relationship between autocorrelations and melodiousness ratings, we calculated additional models for *all poem versions* as recited by the professional speaker, with mean ratings as the dependent variable. The model included the covariate *mean autocorrelation* (together with the fixed effects *acoustic measure* and *textual unit*).

We finally were interested whether autocorrelations would vary dependent on whether the respective poems were set to music or not. For this reason, we calculated a model for the original poems with the dependent variable *mean autocorrelation* (across stanzas) and the fixed effect *set to music* (1 = yes, 0 = no). We additionally ran mixed-effects logistic regressions ([37]) with *set to music* as the dependent variable and *autocorrelation* (across stanzas) as the fixed effect.

## Results

### Original poems: Correlations between autocorrelations scores and subjective melodious ratings

**Fig 3:**
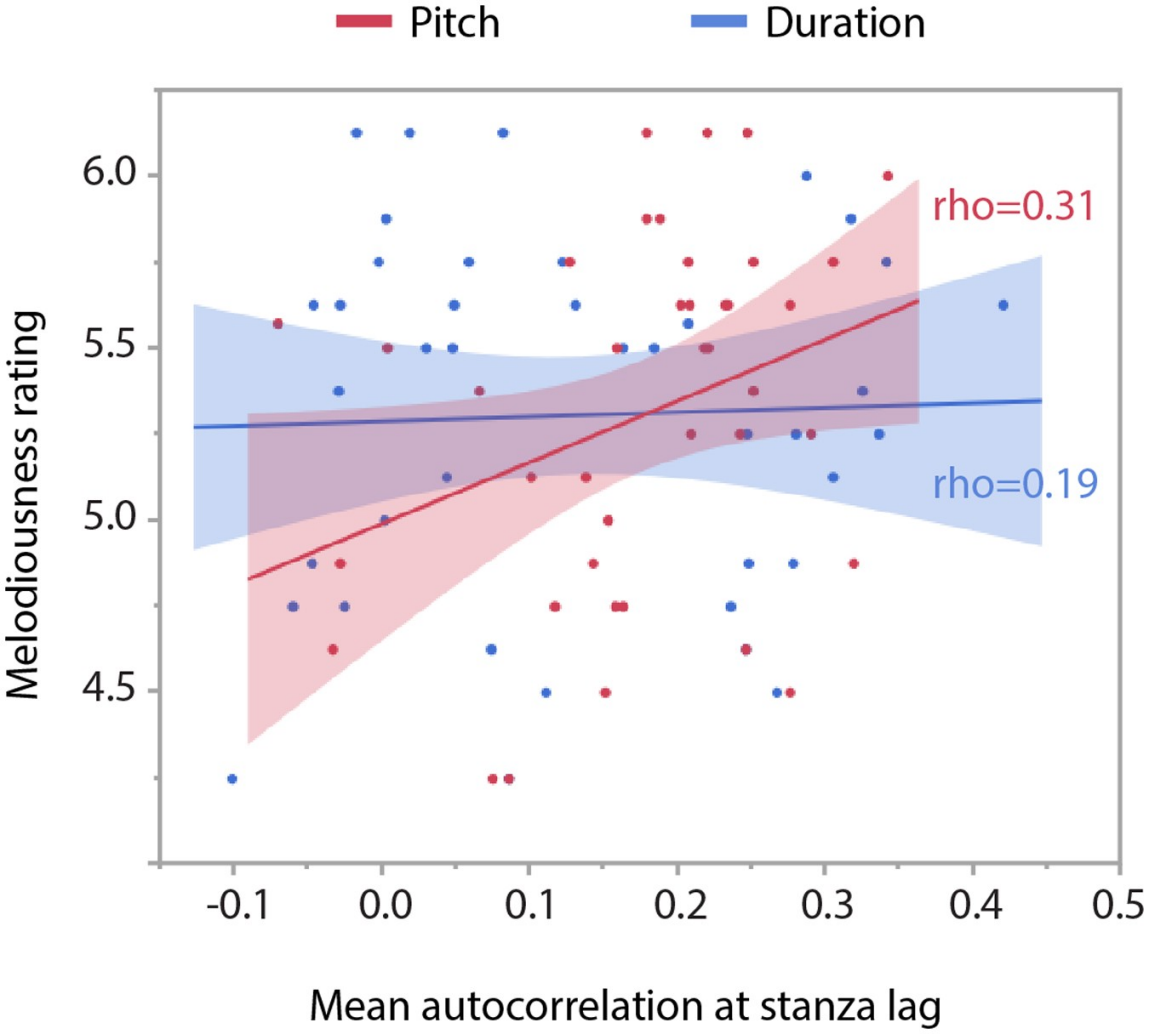
Correlations of melodiousness ratings with stanza-lag autocorrelations, based on pitch (red) and on duration (blue), for the 40 original poems, recited by the professional speaker. Pitch-based autocorrelations were significantly correlated with melodiousness ratings.

Focusing on the original poems only, we first correlated the mean melodiousness ratings obtained from the four different data sets involving four different groups of participants (see “Participants”) with the mean autocorrelation scores of pitch and duration across stanzas as extracted from the rendition of these poems by a professional speaker. There was a significant correlation effect for pitch-based autocorrelations (rho = 0.31, *t* = 2.00, *p* < 0.05) but not for duration-based autocorrelations (rho = 0.19, *t* = 1.32, *p* = 0.20, see Fig 3). Importantly, thus, our statistical measure of the melodiousness of speech captures objective differences of the acoustic rendition of different poems that are predictive of the subjective impressions of melodiousness during listening to these poems.

We further analyzed whether pitch- and duration autocorrelation values varied depending on the respective meter of the original poems in our corpus. Performing a two-sample t-test, we first compared the mean ratings obtained for the iambic poems (N = 27) with those obtained for the trochaic ones (N = 13). The test revealed no significant difference in melodiousness ratings for the two groups of poems (*t* = 0.61, *p* = 0.54). Next, we examined whether there was an interaction between meter in general (be it iambic or trochaic) and acoustic property (pitch, duration) with respect to the mean autocorrelations across stanzas. The corresponding model did not show a significant interaction (*F*(1,38) = 0.74, *p* = 0.39) and hence no effect of meter (*F*(1,38) = 0.01, *p* = 0.92). That is, neither melodiousness ratings nor autocorrelation scores depend on the meter of the original poems.

### Effects of poem modification and autocorrelation lag

#### Professional speaker and synthetic voices

**Fig 4:**
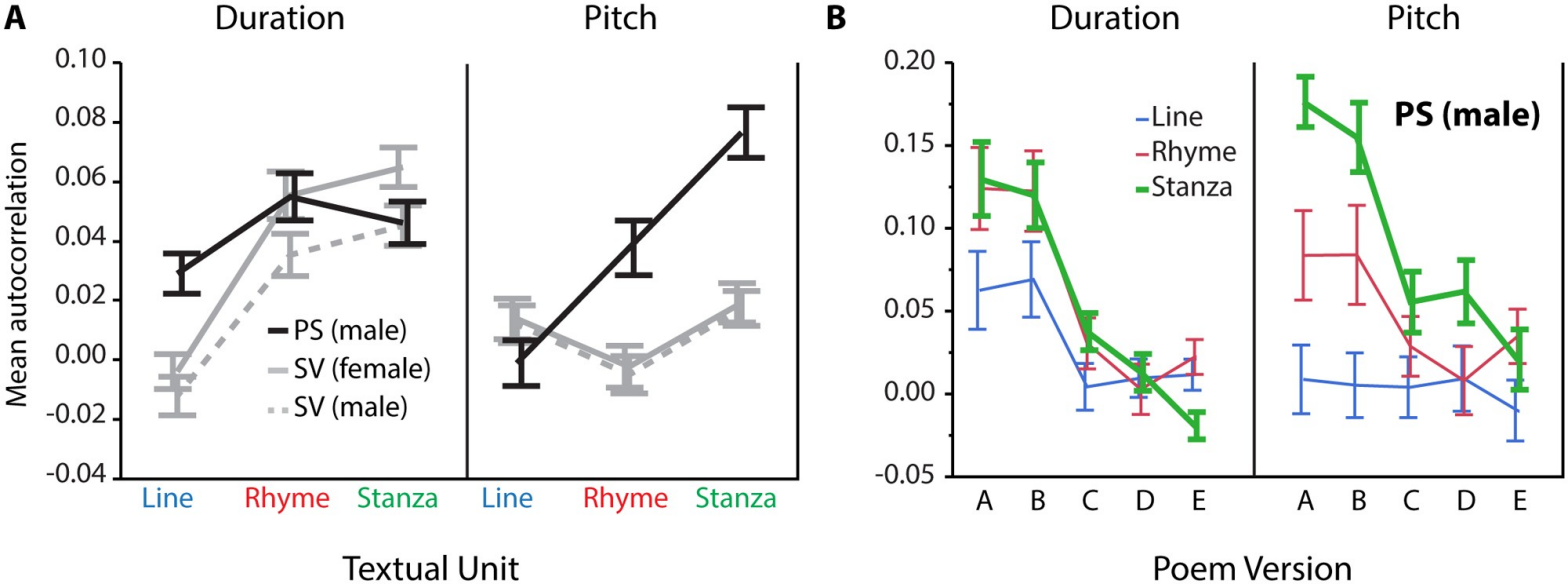
Illustration of textual unit and poem version effects on pitch- and duration-based autocorrelations. A. The scaling of autocorrelations with textual unit (all lines<rhyming lines only<stanzas) crucially depended on acoustic dimension and on speaker (PS: professional speaker, SV: synthetic voice). B. Illustration of the poem version effect for the professional speaker. The strongest effect is seen for pitch-based autocorrelations across stanzas. Error bars indicate standard errors of the mean.

Mean autocorrelations decreased as a function of *poem version* (*F*(4,3471) = 71.65, *p* < 0.001): values were highest for the original versions (A) and lowest for the most modified versions (E). This effect interacted with *textual unit* (*F*(8,3471) = 7.98, *p* < 0.001): modifications affected most strongly autocorrelations computed across stanzas (Fig 4B). The analysis also yielded a main effect of *speaker* (*F*(2,3471) = 25.97, *p* < 0.001), with higher autocorrelations for the professional speaker than for either of the synthetic voices. The main effect of *textual unit* (*F*(2,3471) = 55.12, *p* < 0.001) reflected the following scaling of autocorrelations: all lines<rhyming lines only<stanzas. This effect crucially depended on *acoustic measure dimension* (*textual unit* x *acoustic measure*: *F*(2,3471) = 16.78, *p* < 0.001) and was further influenced by *speaker* (*speaker* x *acoustic measure* x *textual unit*: *F*(4,3471) = 14.12, *p* < 0.001). Notably, the scaling all lines<rhyming lines only<stanzas particularly held for pitch and for the professional speaker (Fig 4A). No other main effects or interactions depended on *speaker* (all *F*s < 2, *p* > 0.19).

#### Nonprofessional speakers

**Fig 5:**
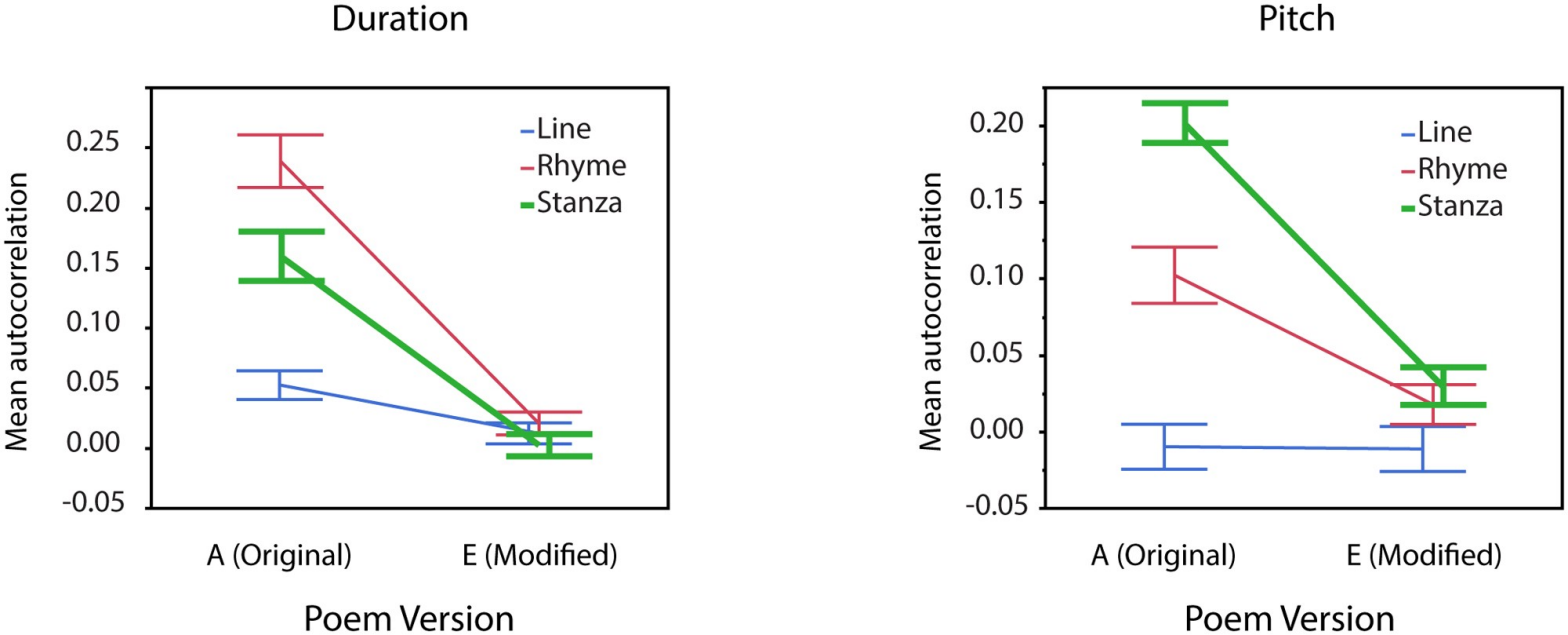
Modification effect on autocorrelations at the textual unit of all verse lines (blue), rhyming lines only (red) and stanzas (green), obtained from renditions by nonprofessional speakers (N = 10). Overall, autocorrelations are higher for original than for modified poems, but differ depending on acoustic dimension (pitch or duration) and textual unit (all lines, rhyming lines only, stanzas). Error bars indicate standard errors of the mean.

Poem modification led to decreased autocorrelation values (*F*(1,833) = 191.40, *p* < 0.001), and autocorrelation values scaled with *textual unit* as in the previous analysis (i.e., all lines<rhyming lines only<stanzas; main effect *textual unit*: *F*(1,833) = 49.67, *p* < 0.001). The significant *textual unit* x *acoustic measure* interaction (*F*(2,833) = 14.87, *p* < 0.001) revealed that this scaling order only held for pitch. Post hoc analyses showed that, for pitch, the stanzas showed larger autocorrelation values than the rhyming lines (*z* = 3.96, *p* < 0.01). Inversely, for duration, the stanzas showed smaller autocorrelation values than the rhyming lines (*z* = -3.46, *p* < 0.01). This effect further depended on *poem version* and was driven by the original poems (significant interaction *textual unit* x *acoustic measure* x *poem version*: *F*(2,833) = 7.16, *p* < 0.001). The stanza>rhyming lines relation held for pitch (*z* = 4.53, *p* < 0.001), and the rhyming lines>stanzas relation held for duration (*z* = -3.60, *p* < 0.001), but no differences were found for the modified poems (pitch: *z* = 0.75, *p* = 0.91; duration: *z* = −1.12, *p* = 0.53; Fig 5). The main effects of *speaker* (*F*(9,833) = 2.93, *p* < 0.05) revealed speaker-dependent differences in the autocorrelations. Importantly, the effect of *speaker* did not show significant interactions with any of the other effects (all *F*s < 2, *p* > 0.12).

### Correlations of autocorrelations scores and subjective melodiousness ratings across poem modifications

Correlating the melodiousness ratings obtained for all 200 poem versions (40 poems in five variants each) with the autocorrelation scores of each of these poems versions revealed that melodiousness ratings strongly depended on the poems’ *modification* (*F*(4,156) = 42.41, *p* < 0.001), with decreasing melodiousness ratings for increasing levels of *poem modification* (see Fig 2). The linear decrease of melodiousness ratings with increasing modification [coding version A as 0, version B and C as 1, and version D and E as 2 and 3 respectively] is substantiated by a significant Spearman correlation (rho = −0.59, *p* < 0.001; based on the mean melodiousness ratings per poem version).

**Fig 6:**
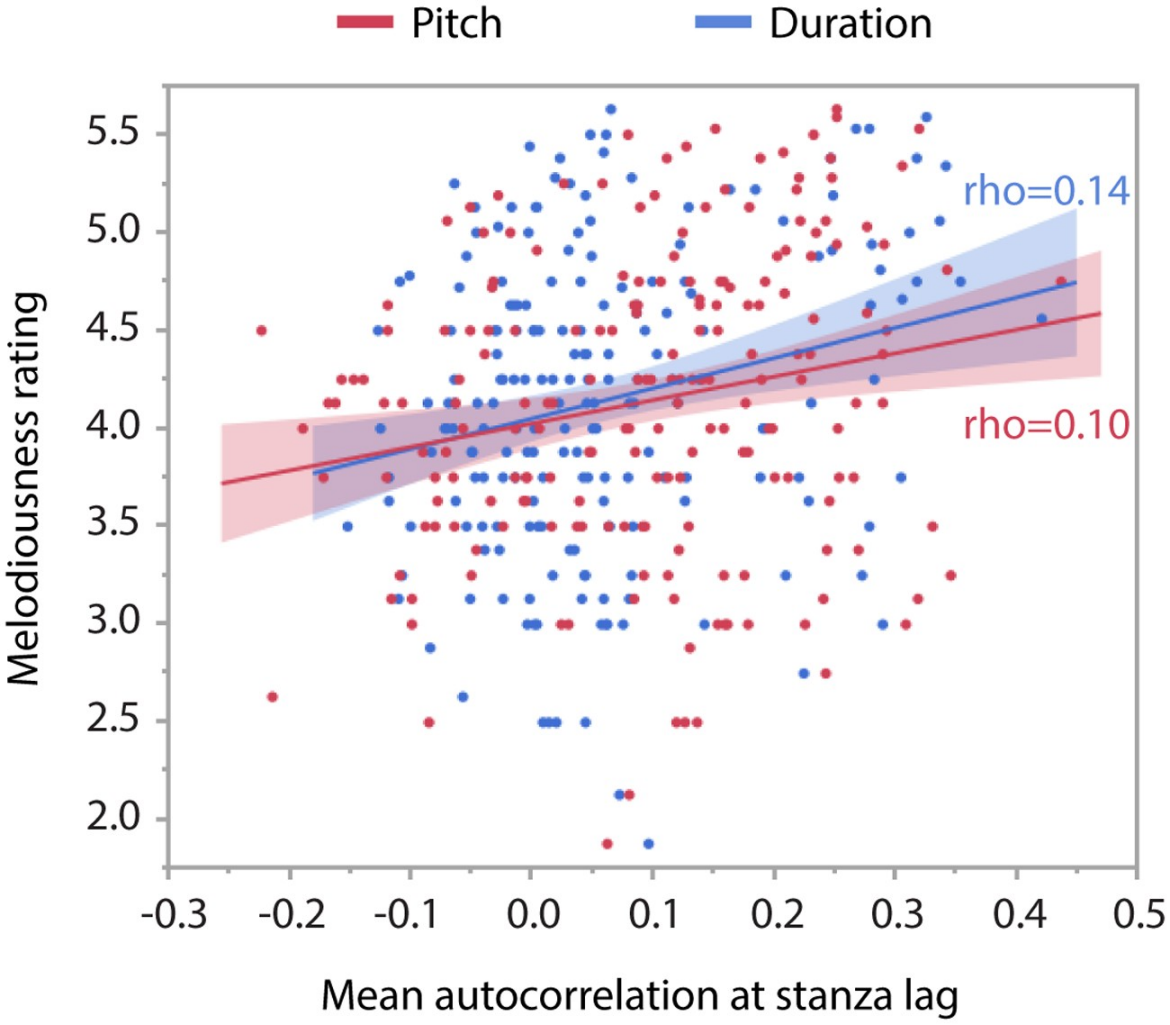
Overall correlations between melodiousness ratings and mean autocorrelations at stanza lag, plotted separately for pitch-based autocorrelations (red) and duration-based autocorrelations (blue). These correlations involve all poem versions.

In the model comprising the mean autocorrelation values of all poem versions as fixed effect, there was a significant interaction of textual unit and mean autocorrelation (*F*(2,1104) = 2.32, *p* < 0.05) that depended on poem modification, as seen in the three-way interaction of textual unit x poem modification x mean autocorrelation (*F*(8,1104) = 2.23, *p* < 0.05). The decomposition of these interactions revealed that mean autocorrelations at line-lags never correlated with melodiousness ratings (i.e. independent of modification, rho = 0.06, *t* = 1.22, *p* = 0.23), whereas mean autocorrelations at rhyme-lags (rho = 0.11, *t* = 2.04, *p* < 0.05) and even more so at stanza-lags (rho = 0.21, *t* = 4.34, *p* < 0.01) correlated positively with melodiousness ratings, with the original poem version showing the by far strongest correlation with the autocorrelation measure. Thus, we observe an overall positive correlation of mean autocorrelations and melodiousness ratings relatively independent of modification. This finding indicates that our statistical measure of melodiousness captures statistical differences of the phonetic signal that correlate with perceptual differences not just for the prototypical rhymed and metered poems but likewise for their far less prototypical versions. Although not substantiated by a significant interaction, we looked at correlations between ratings and autocorrelations at stanza-lags separately for pitch- and duration-based autocorrelations (Fig 6). These correlations proved to be significant for both pitch (rho = 0.10, *p* < 0.05) and duration (rho = 0.14, *p* < 0.01).

Whereas the removal of ongoing meter required systematic changes of the wording or at least the word order throughout all lines of the poems, the other three modifications affected only individual words and were altogether very subtle. Given that our data also show substantial individual variance regarding the ratings for all versions, it is fairly remarkable that we did find a significant correlation between autocorrelation scores and melodiousness ratings for *all* poem versions. Anticipating that this correlation should be far more pronounced when looking at the end points of the experimental modifications only, we computed an additional correlation analysis for versions A and E only. Results strongly confirmed this expectation: The correlations for both pitch (rho = 0.22, *p* < 0.05) and duration (rho = 0.36, *p* < 0.05) were highly significant when looking at the pooled data from versions A and E. By contrast, when looking at the pooled data from versions B, C, and D, correlations did not reach significance, neither for pitch (rho = 0.05, *p* = 0.62) nor for duration (rho = −0.03, *p* = 0.74).

### Autocorrelations and musical settings

**Fig 7:**
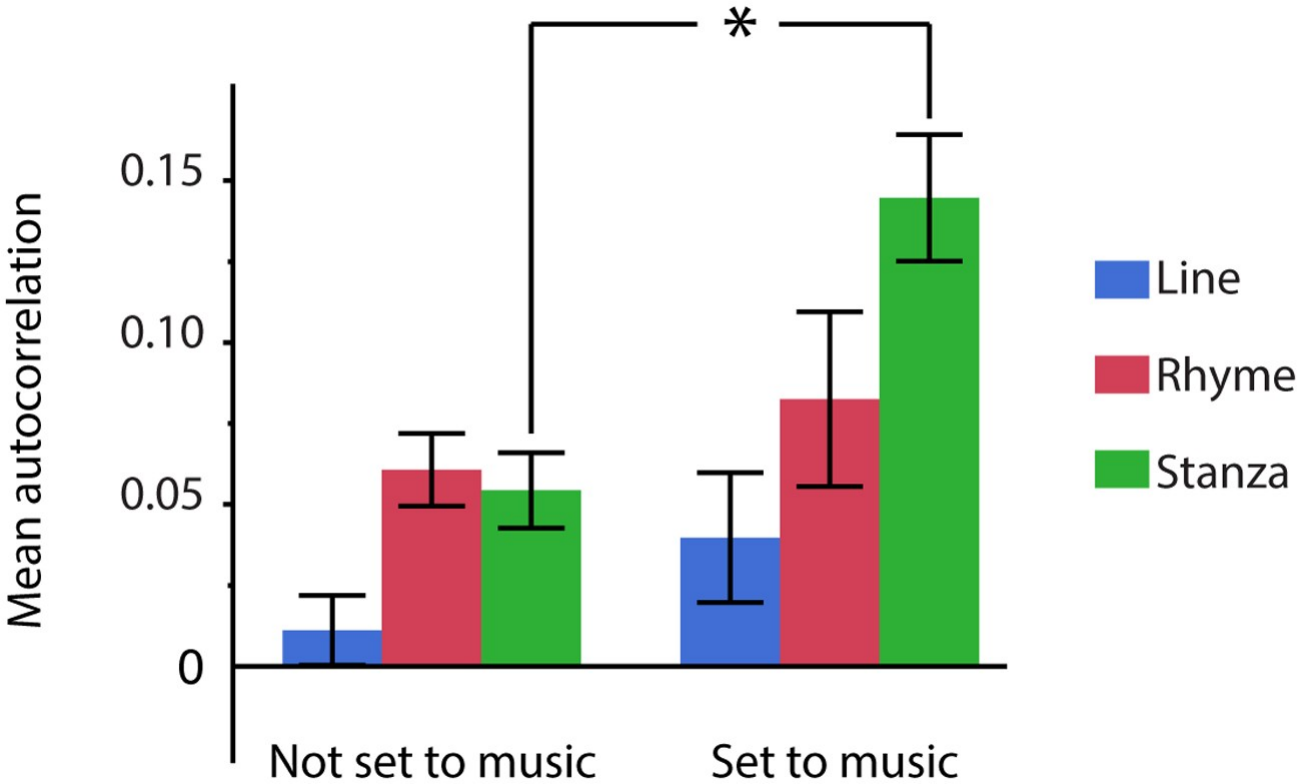
Dependency of pitch autocorrelations and musical settings. Autocorrelation values are higher for poems that have been set to music than for poems that have not been set to music. This particularly holds for autocorrelations across stanzas. Error bars indicate the standard error of the mean.

Whether or not a poem has been set to music (*musical setting* 1 or 0) correlates with mean autocorrelations (*F*(1,399) = 18.68, *p* < 0.001). This effect differs depending on the *textual unit* (all individual lines, rhyming lines only, or stanzas; interaction *musical setting* x *textual unit*: *F*(2,427) = 2.90, *p* = 0.05). Poems set to music particularly show higher autocorrelations across stanzas (*z* = 4.50, *p* < 0.001, Fig 7; all other comparisons *z* < 2, *p* > 0.19). A mixed-effects logistic model further confirmed that musical settings are predicted by overall autocorrelation across stanzas (*z* = 3.40, *p* < 0.001). Again, we found a stronger predictive effect for pitch (*z* = 2.84, *p* < 0.01) than for duration (*z* = 2.27, *p* < 0.05). Notably, this finding was obtained based solely on the original poems, and hence independent of any experimental modification.

## Discussion

The most seminal finding of our study is that pitch contours of original poems show a highly recurrent and largely speaker-independent structure across *stanzas*. These recurrent pitch contours are an important higher order parallelistic feature that had previously escaped attention both in linguistics and in literary scholarship. Crucially, the quantitative measure of pitch recurrence across stanzas correlated significantly with listeners’ melodiousness ratings, lending strong support to our hypothesis that relevant and distinctive dimensions of melodic contour in spoken poetry can indeed be approximatively captured by a fairly simple and abstract autocorrelations measure. Moreover, the fact that mean subjective melodiousness ratings for the 40 original poems nearly converged for four independent groups of participants, is already in itself a remarkable finding that strongly hints at some objective correlate of these ratings.

As anticipated, pitch and duration autocorrelations decreased as other parallelistic properties of the poems were experimentally removed. Importantly, across all poem versions, higher degrees of pitch recurrences predict higher subjective melodiousness ratings, and they do so already for the original (unmodified) poems only, independent of any experimental modification we performed. Duration recurrences also predict melodiousness ratings when analyzed across all poem versions, but not when only the original poems are considered. The absence of an effect of duration autocorrelation for the original poems may suggest that the construct of melody in spoken poetry is mainly based on discrete pitches, in close resemblance to music.

Overall, this pattern of finding suggests that our measure of melodic recurrence––autocorrelations of pitch and duration––is indeed not only suited for analyzing classical metered and rhymed poems of the type that was preeminent in 19^th^ century Europe. From a technical point of view, the measure can easily be applied to all types of speech. It is widely acknowledged that every type of speech has an inherent rhythm and beat (e.g. [20,21]), i.e. a (quasi)-regular distribution of phonetic (speech sound and prosodic) features in time. Our measure is well-suited to capture these distributions in future research. Given that we found an overall positive correlation of melodiousness ratings with autocorrelations across different levels of modifications (i.e. relatively independent of meter and rhyme), we expect that the perceptual consequences of melodic recurrence also hold beyond poetry, albeit to a lesser degree.

Furthermore, pitch recurrences were also predictive of whether or not specific poems were set to music. Our study is thus the first to operationalize the phantom of a “song”-like poetic speech melody of spoken poems by recourse to a measure that can quantify it. It is also the first to empirically illustrate the powerful effects poetic speech melody can exert on the aesthetic evaluation of poetry by nonprofessional listeners as well as on decisions of composers to set particular poems to music.

At first sight, consistencies in syllable pitch (and duration) structure within larger constituents of spoken language may not seem surprising, as previous research on intonation contours and linguistic rhythm (e.g. [38,39]) has revealed that phrase endings are prosodically marked, in that, for instance, pitches yield a downward movement or a falling contour, and that this prosodic marking may co-occur with phrase-final lengthening ([40,41]).

Furthermore, prosody is differently treated in trochaic and iambic meter. The inverse strong-weak and weak-strong patterning of syllables characterizing trochees and iambs is accompanied by prosodic cues that are analogously interpreted across different languages and even mark an important distinction for a nonhuman species ([42]). Stated in the so-called *iambic/trochaic law* ([43,44]), the strong-weak patterning in trochees is brought about by higher intensity and pitch in strong and lower intensity and pitch in weak syllables. On the other hand, the weak-strong patterning in iambs corresponds to a difference in syllable duration, with a relatively short syllable in weak position and a relatively long syllable in strong position.

Thus, to a certain degree, metrical structure alone already supports a regular patterning of pitches and durations. This may certainly be one factor that explains why original poems show high autocorrelations based on these measures. However, this explains neither the correlations of pitch (and partly also of duration) autocorrelations with melodiousness ratings, nor the relationship between pitch structure and the likelihood of a poem being set to music. Since a bit more than two thirds of our original poems feature iambic and the remaining ones have trochaic meter, the aforementioned duration emphasis of iambs should have prevailed over the pitch emphasis of trochees and should in sum total have resulted in a stronger duration than pitch effect. However, we here report precisely the opposite, namely, a stronger predictive power of the pitch autocorrelations. Moreover, we did not find any significant correlations between autocorrelations values and meter (be it iambic or trochaic).

We therefore suggest that the construct of melody in spoken poetry is neither a mere phantom implicitly endorsed by the longstanding tradition to call poems “songs” nor a mere side effect of metrical structure. Rather, it is a measurable, quantifiable entity of its own that explains effects that are not otherwise predictable by existing paradigms and methods of analyzing linguistic prosody.

To be sure, we are fully aware that our analyses cannot provide a full theory of melody or melodic features (for research in this direction, see e.g. [45,46]). Clearly, the autocorrelations scores are exclusively linked to degrees of repetition and not to specific harmonic qualities of the tone sequences. However, for all its abstractness, the predictive power of this statistical measure both for subjectively perceived melodiousness and for decisions of composers to set specific poems to music strongly suggests that the measure does have a bearing on genuine aesthetic perception.

We certainly acknowledge that melody in music and melody in speech differ in certain aspects. For instance, the pitch range in speech is far narrower than in musical melody ([47,48]). Nevertheless, the proposed measure of pitch- and duration-based autocorrelations appears to be a fruitful measure that at least approximately captures melodic properties of both music and language.

As predicted by our theoretical considerations, similar pitch sequences were most prominently found across stanzas. After all, it is primarily the stanza pattern that is consistently repeated in poems, whereas individual lines vary frequently in the number of syllables. Since recurrent meter and rhyme patterns have been shown to enhance prosodic fluency ([14]), it is likely that recurrent melodic contours also contribute to such parallelism-driven fluency effects which, in turn, enhance aesthetic appreciation ([49]). In fact, we propose that our results can largely be explained by reference to the *ease-of-processing* hypothesis of aesthetic liking.

Crucially, the stanza effect in our study turned out to be consistently independent of the speaker. It was most pronounced when poems were recited by humans (professional or nonprofessional). Surprisingly––and highlighting the independence of poetic speech melody from the actual rendition by any speaker and hence its strong reliance on an inherent textual property––, even the poem versions that were recited by synthetic voices confirmed the melody effect at the stanza level. The ratings of perceived melodiousness likewise correlated most strongly with stanza-based pitch and duration autocorrelations.

The strong effect of the poem modifications on melodiousness as spontaneously rated by non-expert listeners suggests that poetry recipients are highly sensitive to perceiving multiple co-occurring and strongly interacting parallelistic patterns at a time and that they are also capable of rapidly integrating these patterns into a complex percept. Such automatic detection and integration of multiple optional patterns of poetic parallelism can be conceived as an analogue to the low-level perception of multiple symmetries and other autocorrelations in complex visual aesthetics ([50]).

Moreover, parallelistic patterning has been shown to enhance the memorability of poetic language ([51]). As genuine musical melodies clearly support the memorability of the lyrics underlying them, it is worth investigating the extent to which poetic speech melody also increases verbatim recall and potentially also the privileged storage of poems or other texts in memory ([51]).

Finally, our analyses of poetic speech melody reveal a hitherto unknown reason for why some poems have been set to music while others have not: the higher the degree of pitch recurrences of corresponding syllables across the stanzas of a given poem, the higher the likelihood that it has been set to music. Thus, our findings provide an empirical basis for the view that melodic aspects of poetry are inherent properties of the verbal material itself ([52]), and that an intuitive awareness of these properties seems to guide composers in finding the “right” musical melody ([53]).

Regarding the relationship of linguistic prosody to music, our methods advance attempts by Halle and Lerdahl [54] to introduce generative methods for “text setting” (i.e., setting texts to music) by capitalizing on the fact that linguistic and musical prosody are similar ([9,55,56]). Our findings––particularly the predictive power of stanza-related autocorrelations for musical settings––suggest that composers are not only aware of the relationship between linguistic prosody and music, but also have the skills to implement the transformation that Halle and Lerdahl [54] described.

In his novel *War and Peace* ([57]), Tolstoy evocatively refers to this notion of a genuine inherent melodiousness of poetic language: “The uncle sang […] in the full and naive conviction that the whole meaning of a song lies in the words only and that the melody comes of itself, and that […it] exists only as a bond between the words.” In the end, this is exactly our finding: there is, indeed, a melody emerging from the words only––from the process of selecting and combining them––, and this auto-emergent melody bestows an additional musical coherence on the entire word sequence.

We certainly acknowledge that the relation between language and music is unlikely always to be as straightforward as our analyses of a specific type of poetry suggest. For instance, prose, too, can be set to music (operatic recitative), and some texts set to music may not show pronounced musical contours (monotonic chanting in certain religious traditions). Furthermore, many poems do not feature any sustained rhyme and/or metrical pattern and/or no stanza structure (e.g. [24]). However, even in these cases, our measure of pitch and duration recurrence may well be able to shed light on the relations between intrinsic language-dependent intonation and musical melody as well as between intrinsic linguistic rhythm and musical beat.

### Summary

Summing up, our data strongly support the notion that the spontaneous recognition of recurrent melodic patterns extends well beyond music proper and the expectations of tonal harmony with which they are associated in music. Our study shows that spoken texts show, in their compositional entirety, a genuine and consistent patterning of recurrent pitch and duration contours, that a melodiousness of this type can be captured statistically using the same metrics across acoustic domains, and that recipients readily project their intuitive percept of inherent language-based melody onto melodiousness ratings in a way that is highly consistent with our statistical measure of melodiousness. Thus, our study turns the phantom of a poetic speech melody in spoken texts into a non-metaphorical, unquestionably real and measurable entity.

Finally, both classical ([58]) and modern poetics ([13]) suggest that poetic language is only gradually and not categorically different from ordinary language. Seen in this light, the perceptual sensitivity to poetic speech melody that we report in this study is unlikely to be exclusively acquired through repeated exposure to poetry. Therefore, we expect the construct we introduce in this study to be helpful in making progress on other issues that have thus far remained fairly elusive, specifically melody-like structures in rhetorical speeches, spoken religious liturgy and other types of ritual language that are rich in parallelistic structures. The measure may also be helpful for making progress on the difficult issue of “prose rhythm” ([59]), provided that in prose, too, higher order recursive structures can be identified that are analogues, if only less rigid ones, to the lines and stanzas of poetry, and hence can serve as reference units for more fine-grained autocorrelation analyses.
